# Home-based high tone therapy may alleviate chemotherapy-induced neuropathic symptoms in patients with colorectal cancer: A randomized double-blind placebo-controlled pilot evaluation

**DOI:** 10.1007/s00520-024-08331-7

**Published:** 2024-01-27

**Authors:** Robert Wakolbinger-Habel, Matthias Reinweber, Mahmoud Alakraa, Ingrid Riener, Brigitte Elisabeth Scheffold, Krisztina Racz, Flonza Selimi, Claudia Straub, Jakob Jauker, Walter Bily, Dora Niedersüß-Beke, Karl Mayrhofer, Tatjana Paternostro-Sluga

**Affiliations:** 1 Department of Physical and Rehabilitation Medicine (PRM), Vienna Healthcare Group–Clinic Donaustadt, Langobardenstraße 122, 1220 Vienna, Austria; 2 External Lecturer, Medical University of Vienna, Währinger Gürtel 18-20, 1090 Vienna, Austria; 3 Vienna Healthcare Group–Directorate General, Thomas-Klestil-Platz 7, 1030 Vienna, Austria; 4 Medical Department II, Vienna Healthcare Group–Clinic Donaustadt, Langobardenstraße 122, 1220 Vienna, Austria; 5 Department of Physical and Rehabilitation Medicine (PRM), Vienna Healthcare Group–Clinic Ottakring, Montleartstraße 37, 1160 Vienna, Austria; 6 Medical Department I, Vienna Healthcare Group - Clinic Ottakring, Montleartstraße 37, 1160 Vienna, Austria

**Keywords:** Chemotherapy-induced peripheral neuropathy, Physical medicine, Rehabilitation, High tone therapy

## Abstract

**Background:**

Most oncologic patients receiving chemotherapy suffer from neuropathy, which not only severely affects quality of life but also may lead to chemotherapy dose reductions or even discontinuation of cancer therapy. Still, it is difficult to sufficiently control these symptoms with the currently available pharmacological treatments. High tone therapy was reported to be an effective option for neuropathies due to different etiologies. However, to date, there are no studies on high tone therapy in patients with chemotherapy-induced peripheral neuropathy.

**Methods:**

This randomized, double-blind, and placebo-controlled two-center study was conducted at the Departments of Physical and Rehabilitation Medicine at the Clinics Donaustadt and Ottakring, Vienna, Austria. Patients with histologically verified colorectal carcinoma treated with a platin derivate and neuropathic symptoms were invited to participate. High tone therapy took place in a home-based setting using the HiToP 191 PNP ® or placebo device for three weeks. Neuropathic symptoms and quality of life were assessed via questionnaires. After the follow-up examination, an opt-in was offered to the patients in the placebo group in terms of an open-label treatment with a verum HiToP PNP ® device.

In addition, patients with chemotherapy-induced peripheral neuropathy due to various malignant diseases were treated in an open-label setting reflecting a clinical application observation. These patients are reported as a separate group.

**Results:**

In the verum group, there was a significant reduction of paresthesias and mental stress due to paresthesias from baseline until end of therapy, compared to placebo. These findings were observed in the opt-in subgroup, as well. In the open-label clinical application observation group, intensity and mental stress due to paresthesia, pain, cramps, and intensity of tightness/pressure were significantly lower at the end of therapy, compared to baseline.

**Conclusions:**

Home-based high tone therapy brought about a significant alleviation in paresthesias and mental stress due to paresthesias in the verum but not the placebo group. In the clinical application observation, a significant alleviation in several further neuropathic symptoms was seen.

**Trial registration:**

This study was registered at clinicaltrials.gov (NCT06048471, 03/02/2020).

**Supplementary Information:**

The online version contains supplementary material available at 10.1007/s00520-024-08331-7.

## Background

Neuropathy means the damage of peripheral nerves due to various conditions like chronic diseases, injury, or as a side effect of specific medications. Chemotherapy-induced peripheral neuropathy (CIPN) is a frequent side effect of antineoplastic medication, which occurs in approximately two of three patients [32]. CIPN does not only limit the quality of life due to neuropathic symptoms but also may lead to dose reductions or premature discontinuation of therapy and thus to suboptimal cancer treatment.

Patients with CIPN suffer from sensory disturbances as tingling, numbness, burning pain or sleep disturbances. Even though numerous drugs are available, it is still difficult to sufficiently control these symptoms [11, 27, 33] and potential side effects need to be considered [22, 31]. According to the ASCO and ESMO guidelines, only for duloxetine there is level I evidence and to date, no recommendations for transcutaneous electric stimulation (TENS) can be made [13, 17].

High tone therapy could be an effective treatment for neuropathic symptoms. It delivers medium frequency alternating current. In detail, the carrier frequency lies between 4 and 33 kHz and is modulated at a defined frequency of 20 Hz. This procedure is called SimulFAM® (simultaneous frequency amplitude modulation). The mechanisms of action are not clear yet. Potential mechanisms are spinal stimulation, promotion of attachment and differentiation of hematopoietic stem cells, neovascularization, stronger microcirculation, increase in blood flow, and improved endothelial function [8, 9].

Previous studies observed promising results in diabetics [8, 9, 15, 30] and chronic kidney disease (CKD) patients [16, 34].

To date, there are no studies on high tone therapy in patients with CIPN. As platin derivates exhibit neuropathic symptoms very frequently (75%) [3] and are used as a standard therapy for colorectal cancer, this pilot evaluation should investigate this specific group of patients.

The aim of this study was to test if high tone therapy brings about a stronger decrease in neuropathic symptoms in patients with colorectal cancer, compared to placebo.

## Methods

### Design

This two-center pilot study was conducted at the Departments of Physical and Rehabilitation Medicine (PRM) of the Clinic Donaustadt and the Clinic Ottakring, Vienna, Austria. This study was randomized, double-blind, and placebo-controlled.

### Subjects

Female and male patients of the Departments of Oncology were screened for eligibility and invited to participate in the study. The inclusion and exclusion criteria were checked at the Departments of PRM. This study was approved by the local Ethics Committee (Ethikkommission der Stadt Wien, EK 19–124-0619, Oct 31st 2019) and conducted in accordance with the Declaration of Helsinki. Prior to any procedure, written informed consent was obtained.

Inclusion criteria:

- Patients with histologically verified colorectal cancer and adjuvant treatment with a platin derivate (e.g., cisplatin and oxaliplatin)
- Cumulative dose of at least 3 cycles
- Life expectancy of at least 3 months
- Stable medication (no changes of especially pain medication during the study)
- Eastern Cooperative Oncology Group (ECOG) Performance Status score of 0–2 (that is, the capability to walk and to spend less than 50% of waking hours sitting or lying)
- Ability to walk (with or without aids)
- European Organisation for Research and Treatment of Cancer (EORTC) common toxicity criteria (CTC) peripheral sensory neuropathy grade 1 or 2
- Intensity of paresthesias of 3/10 or higher on the Numeric Rating Scale (NRS)

Exclusion criteria

- Prevalent neuropathy of different etiology
- Serious central-neurological or psychiatric disorder that would interfere with a proper order of the study, according to the judgement of the investigators
- Epilepsy
- Minors or persons unable to give informed consent
- Implanted pacemakers or defibrillators
- Pregnancy
- Wounds in the area to be treated, acute local or systemic infection
- Peripheral arterial occlusive disease > grade 2

### Intervention

The HiToP® 191 PNP (GBO Medizintechnik AG, Rimbach, Germany) is a CE-certified and patented (European Patent 1322379B1) medical product, and to date, no undesirable side effects have been reported.

The high tone therapy does not involve a typical “electricity sensation” but rather only a feeling of muscular contraction in some but not all persons. Some patients do not feel anything during the treatment. Therefore, a placebo-controlled investigation was feasible.

In the placebo group, a placebo device of the same design was used with no current output.

Previous studies observed alleviation of neuropathic symptoms after a relatively short treatment period [8, 9, 15, 30]. However, most of these studies had no control group and therefore, the data on the effect of high tone therapy is weak.

The home-based treatment phase in our study lasted three weeks, while the first session took place in the Departments of PRM to instruct the patients in the handling of the device. One treatment session lasted 60 min. The minimum number of treatment sessions to be completed was 5 out of 7 days. The device saves minutes of treatment and therefore offered the clinicians to monitor the participants’ compliance.

In patients with neuropathic symptoms in the lower extremities, the electrodes were placed onto the calves and soles. In case of symptoms in the upper extremities, they were placed onto the upper arm and palm.

### Assessments

Assessments were carried out at baseline (before the first treatment session), after the first treatment week, at the end of the treatment phase and follow-up two weeks after the end of the treatment phase.

The baseline assessment included a test of pallesthesia [26]. In this examination, certain anatomical localizations were stimulated with a tuning fork (i.e., the dorsum of the interphalangeal joint of the hallux and the medial malleolus). Several studies reported that this test not only detects peripheral neuropathy accurately but also seems to be superior to the monofilament test [21, 25]. Moreover, normative values for several age groups have been published [20].

In addition, full medical and social history were obtained, including demographic data, previous conditions as well as the course and treatment of the current cancer, duration of neuropathy, and (pain) medication.

The following outcome parameters were determined at all points of time:

- Neuropathic symptoms
  o Numeric rating scale (NRS, 0–10): Paresthesia intensity, mental stress due to paresthesia, pain intensity, mental stress due to pain, tightness/pressure intensity, and mental stress due to tightness/pressure
  o European Organisation for Research and Treatment of Cancer Chemotherapy-Induced Peripheral Neuropathy 20 questionnaire (EORTC CIPN 20): This questionnaire assesses chemotherapy-induced neuropathy with a sensory scale, motor scale as well as an autonomic scale [28] The questionnaire has been used in several studies [4, 7].
  o Neuropathy Symptom Score (NSS): This questionnaire assesses neuropathic symptoms and has been used in several studies [8, 12, 19].
- Quality of life: EORTC Core 30 (EORTC C30) questionnaire: This questionnaire specifically assesses health-related quality of life in cancer patients [6].

### Opt-in

After the last follow-up examination, both the patient and physician were unblinded. Patients in the placebo-group were offered an opt-in, that is, receiving the verum treatment for the same period of three weeks.

### Objectives

The primary objective was to compare the changes in intensity of the paresthesias (NRS) from baseline until the end of therapy between the two patient groups.

The secondary objectives were the further neuropathic symptoms and quality of life.

### Hypothesis

We hypothesized that the mean change in paresthesias from baseline until the end of therapy would differ significantly between the verum and placebo group.

### Sample size calculation

Due to the pilot (explorative) character of the study, no sample size calculation was done.

### Randomization

The allocation was performed according to a randomization protocol designed by the Directorate General, Board Division of Health Care Management. This protocol was based on online randomization.

### Blinding

To ensure a double-blind procedure, the participants were supervised by two physicians per center. Physician one performed the assessments and physician two managed the randomization and instruction in the handling of the device. All subjects were informed that they may feel tingling or muscle contraction during the treatment but also about the possibility of not feeling any sensations. Subjects were required not to tell physician one if they felt tingling or contractions.

### Statistics

For comparison of verum and placebo groups, a repeated measures analysis of variance is used. A Box’s *M* test for equivalence of covariance matrices is used to test if covariances are homogenous. Mauchly’s sphericity test is conducted to validate equal variances among the differences between all possible pairs of within-subject conditions. To avoid alpha-error accumulation a Bonferroni correction is calculated. The Kolmogorov–Smirnov test is used to check the normal distribution of dependent variables. Since all statistical requirements are met, a repeated measures analysis of variance, at 4 times of measurement, is used to test differences within groups (baseline vs. end of therapy) and between groups (placebo vs. verum) with a *p*-value (alpha) of 0.05.

For demographic variables, a *t*-test r chi square test are used for comparison (*p*-value 0.05).

### Open-label group

In addition to the double-blind placebo-controlled RCT, we offered the high tone treatment to a higher number of patients with CIPN due to various malignancies. These patients did not participate in the RCT and were evaluated using exclusively the short NRS questionnaire. However, these patients reflect our daily clinical practice and are therefore reported as a separate open-label group.

## Results

**Fig. 1:**
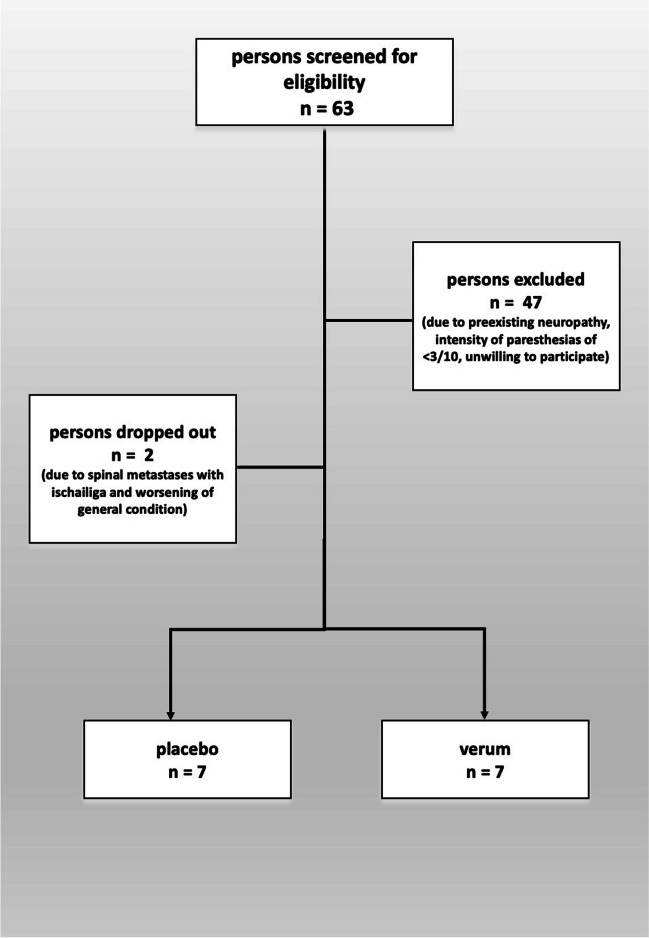
Study flow chart

Sixty-three patients were screened, 47 had to be excluded due to preexisting neuropathy, intensity of paresthesias of < 3/10, or unwillingness of participating in the study. Two patients dropped out due to spinal metastases with ischialgia or worsening of the general condition. Fourteen patients completed the study (Fig. 1).

### Baseline data (Table 1)

**Table 1:** Demography for the placebo and verum group

	Placebo (*n* = 7)	Verum (*n* = 7)	*p*-value
Age (years ± Stdv.)	64 (53–75)	54 (39–57)	0.128
Cancer stage T	3	3	N/A
Cancer stage N	2	1	N/A
Cancer stage M	0	0	N/A
Surgery	7	7	N/A
Time since diagnosis (months ± Stdv.)	20 (1–39)	18 (0–47)	0.924
Type of platin	7 × oxaliplatin	1 × carboplatin, 6 × oxaliplatin	N/A
Chemotherapy schedule	1 × CAPOX, 1 × FOLFIRINOX, 2 × FOLFOX, 1 × ISOFOL, 2 × XELOX	2 × CAPOX, 1 × Docetaxel/Carboplatin, 1 × FOLFIRINOX, 1 × FOLFOX, 2 × XELOX	N/A
Chemotherapy cycles (± Stdv.)	5 (4–6)	7 (4–10)	0.520
Duration of neuropathic symptoms (months ± Stdv.)	7.6 (2.9–15.1)	17.8 (0–41)	0.535
Ongoing chemotherapy (% of patients)	5 (71%)	5 (71%)	N/A
Regular pain medication at baseline (% of patients)	4 (57%)	5 (71%)	N/A

*Stdv* standard deviation; *T* median tumor size (according to TNM classification); *N* median lymphatic node invasion (according TNM classification); *M* metastasis (according TNM classification): no patient had metastases; *Surgery* all patients in both groups had surgery (e.g., hemicolectomy or deep anterior rectal resection); *Time since diagnosis* number of months between the histologic diagnosis of colorectal cancer and the HiToP baseline visit; *Type of platin* all patients (7) in the placebo group had oxaliplatin, and in the verum group, six patients had oxaliplatin and one patient carboplatin; *Chemotherapy cycles* mean number of cycles

Patients in the verum group were slightly younger and had a longer duration of neuropathic symptoms, but these differences were not statistically significant. In most patients in both groups, chemotherapy was ongoing during the study.

### NRS scores of neuropathic symptoms (Table 2)

**Table 2:** Baseline neuropathy symptoms and changes in the numeric rating scale (NRS) from baseline until the end of therapy in the placebo and verum group. The values are provided as absolute numbers ± standard deviation

	Placebo (*n* = 7) baseline	Placebo (*n* = 7) changes at end of therapy	Verum (*n* = 7) baseline	Verum (*n* = 7) changes at end of therapy	*p*-value baseline	*p*-value changes placebo	*p*-value changes verum
Intensity of paresthesias	6.7 (5.2–8.2)	− 1.21 (− 2.73–0.31)	6.3 (3.5–9.0)	− 1.71 (− 3.81–0.39)	n.s	n.s	0.034
Mental stress due to paresthesias	6.1 (4.1–8.2)	− 0.50 (− 2.0–1.0)	6.4 (4.8–8.0)	− 2.43 (− 3.85–0.42)	n.s	n.s	0.006
Intensity of pain	3.0 (﻿0–6.5)	+ 1.21 (− 2.10–4.52)	3.3 (0.3–6.3)	− 0.71 (− 2.31–0.89)	n.s	n.s	n.s
Mental stress due to pain	2.86 (0–6.3)	+ 0.90 (− 2.42–4.22)	3.7 (0.4–7.0)	− 1.43 (− 3.18–0.33)	n.s	n.s	n.s
Intensity of tightness	2.57 (0–5.8)	+ 0.93 (− 2.34–4.20)	1.3 (0–3.5)	+ 0.71 (− 2.62–4.04)	n.s	n.s	n.s
Mental stress due to tightness	3.0 (0–6.8)	+ 0.50 (− 3.45–4.45)	1.9 (0–4.2)	− 0.43 (− 4.74–3.88)	n.s	n.s	n.s
Intensity of cramps	3.0 (0.2–5.8)	− 0.79 (− 3.90–2.32)	1.0 (0–3.2)	0.00 (0–0)	n.s	n.s	n.s
Mental stress due to cramps	2.6 (0–5.3)	− 0.36 (− 3.08–2.36)	1.0 (0–3.2)	0.00 (0 − 0)	n.s	n.s	n.s

The baseline-scores did not differ significantly between the two groups

In the verum group, significant changes in paresthesia intensity and mental stress due to paresthesias were observed, whereas in the placebo group, no significant changes were seen

The baseline intensity of paresthesias was similar in both groups, as were further neuropathic symptoms according to the NRS questionnaire.

In the verum group, there was a significant reduction in the intensity of paresthesias (27%) as well as mental stress due to paresthesias (38%) from baseline until the end of therapy.

**Fig. 2:**
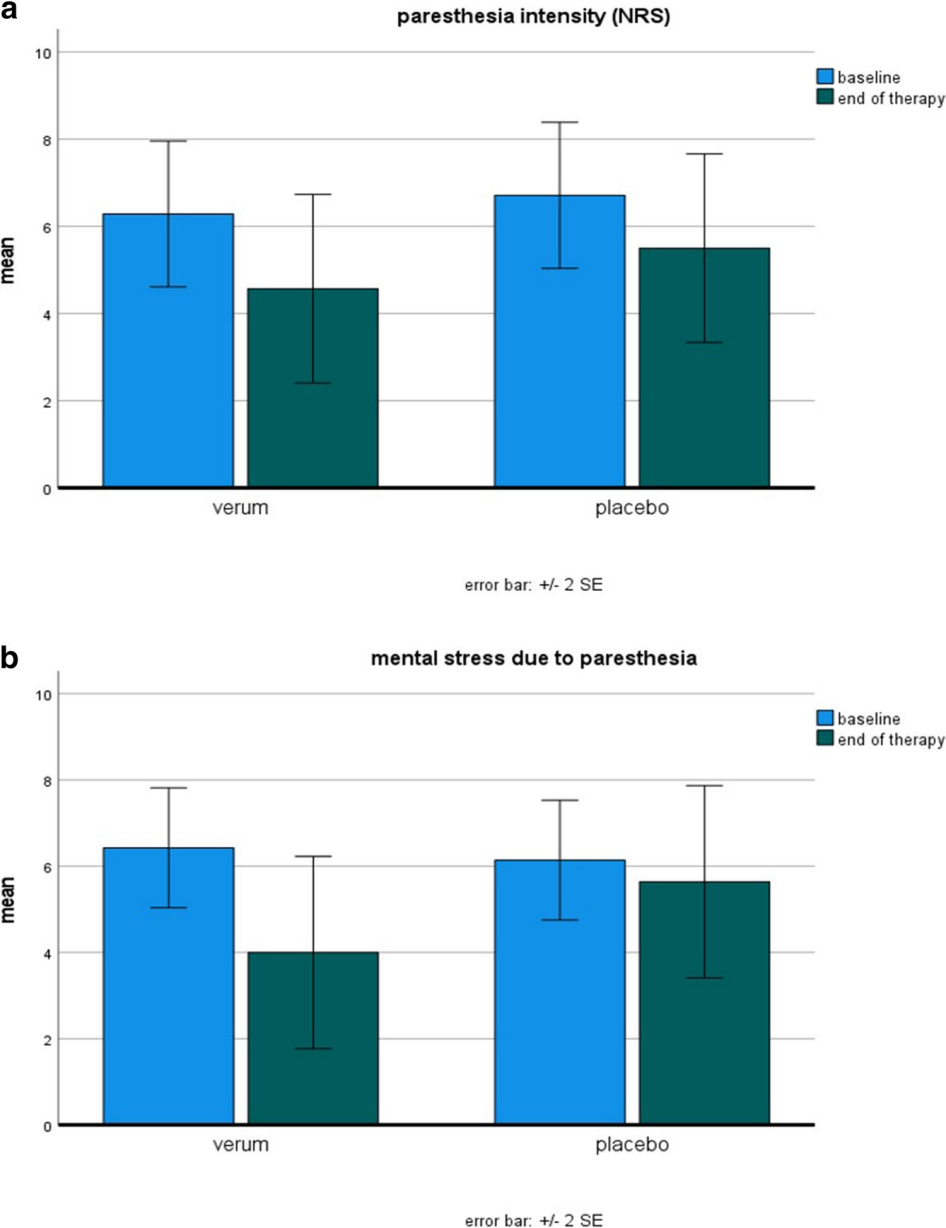
Changes in **A** intensity of paresthesias and **B** mental stress due to paresthesias in the numeric rating scale (NRS) in the placebo and verum group. The values are provided as mean ± 2 × standard error. SE: standard error

In contrast, in the placebo group, no significant changes (18% and 8%, respectively) were observed (Fig. 2).

### EORTC C30, CIPN20, NSS scores (Table 3)

**Table 3:** EORTC C30, EORTC CIPN20 and NSS scores: baseline values are provided for the placebo group and verum group

	Placebo baseline (*n* = 7)	Placebo end of therapy (*n* = 7)	Verum baseline (*n* = 7)	Verum end of therapy (*n* = 7)	*F*-value (baseline vs. end of therapy, interaction with group)	*p*-value
C30 global health status	47 (27–67)	42 (27–56)	36 (19–54)	52 (23–82)	2.662	n.s
C30 physical functioning	76 (56–96)	74 (54–95)	56 (40–72)	64 (36–91)	1.47	n.s
C30 role functioning	49 (15–85)	64 (33–95)	29 (0–60)	50 (13–87)	0.174	n.s
C30 emotional functioning	57 (42–72)	64 (43–86)	54 (36–73)	77 (46–109)	1.804	n.s
C30 cognitive functioning	76 (49–103)	76 (53–99)	61 (26–98)	83 (58–109)	1.529	n.s
C30 social functioning	38 (1–75)	45 (12–78)	40 (10–71)	67 (20–114)	1.864	n.s
C30 fatigue	51 (16–86)	46 (26–66)	60 (30–91)	37 (7–66)	1.228	n.s
C30 nausea/vomiting	12 (0–30)	7 (0–20)	19 (0–23)	0 (0–0)	0.375	n.s
C30 pain	45 (12–78)	50 (26–74)	64 (28–101)	48 (14–82)	1.599	n.s
C30 dyspnea	19 (0–45)	24 (0–55)	29 (0–64)	19 (0–45)	1.227	n.s
C30 insomnia	47 (21–74)	38 (15–61)	38 (15–61)	29 (6–52)	0	n.s
C30 appetite loss	43 (6–80)	38 (0–79)	38 (0–79)	10 (0–26)	1.563	n.s
C30 constipation	5 (0–17)	0 (0–0)	42 (6–80)	33 (6–61)	0.376	n.s
C30 diarrhea	29 (0–59)	38 (8–68)	19 (0–57)	14 (0–41)	1.227	n.s
C30 financial difficulties	19 (0–45)	14 (0–41)	19 (0–26)	0 (0–0)	0.375	n.s
CIPN20 sensory scale	43 (18–70)	45 (23–67)	59 (35–67)	37 (8–65)	1.324	n.s
CIPN20 motor scale	26 (3–48)	29 (8–49)	43 (29–58)	26 (3–48)	2.891	n.s
CIPN20 autonomic scale	32 (15–48)	21 (2–39)	43 (12–74)	25 (0–52)	0.317	n.s
NSS score	6 (5–7)	6 (4–7)	6 (5–7)	7 (5–8)	1.6	n.s

For changes from baseline to the end of therapy in consideration of the interaction with the group, *F*-values are calculated. None of the changes reached statistical significance

No significant changes in the scores of the EORTC C30, EORTC CIPN20 or NSS questionnaire from baseline to the end of therapy under consideration of the group were observed.

### 1st week of therapy and two-week follow-up (supplementary Table 1)

After the first week of therapy, no significant changes were observed.

At the follow-up visit two weeks after the end of therapy, a significant change in mental stress due to paresthesia compared to baseline was observed in the verum group.

### Opt-in group (supplementary Table 2)

In the opt-in group, a significant reduction in the intensity of paresthesias (26%) as well as strong trend for mental stress due to paresthesias (22%) from the start of the opt-in therapy until the end of the opt-in therapy was observed.

### Open-label group (supplementary Table 3)

In the open-label clinical observation group, most NRS symptoms were significantly lower at the end of therapy compared to baseline. The strongest reductions were seen for pain intensity (62%), mental stress due to pain (49%), mental stress due to paresthesias (40%), and paresthesia intensity (38%).

### Adverse events

No adverse events and especially no serious adverse events were reported.

## Discussion

To the authors’ knowledge, this is the first study reporting the effects of high tone therapy on symptoms of chemotherapy-induced peripheral neuropathy. A significant reduction in paresthesia intensity as well as mental stress due to paresthesias was observed in the verum group at the end of therapy compared to baseline, whereas no significant changes were seen in the placebo group.

According to the literature [2, 14], the change of − 1.71 points on the NRS scale from baseline to end of therapy in the verum group is clinically relevant but not the change of − 1.21 points in the placebo group.

Importantly, the treatment was well tolerated; no adverse events and especially no serious adverse events were reported.

A previous work on electric stimulation in patients with chemotherapy-induced peripheral neuropathy investigated transcutaneous electric nerve stimulation (TENS).

Gewandter et al. [7] conducted a one-arm open-label study and reported improvements in pain, tingling, numbness, and cramping after six weeks of treatment. In contrast, Tonezzer et al. observed no differences between the verum and placebo group [36]. However, due to the heterogeneity of the studies on TENS in patients with chemotherapy-induced peripheral neuropathy, a relatively recent systematic review concluded that no strict recommendations could be made [29].

Therefore, not only more studies on classic TENS itself are needed, but also for example comparative head-to-head analyses of TENS and high tone therapy.

After the first week of treatment, no significant changes were observed. These observations indicate that in our population, a certain amount of therapy sessions may be necessary for a significant effect. This contrasts with the studies of Hidmark et al. [8] and Kempf and Martin [15] on patients with diabetic neuropathy, who observed significant changes already after one week of treatment.

At the follow-up visit two weeks after the last treatment session, mental stress due to paresthesia was still lower in the verum group. This suggests a partial sustained effect of high tone therapy, even though compared to baseline, paresthesia intensity was not significantly different anymore.

In the opt-in group, both paresthesia intensity and mental stress due to paresthesia were significantly lower at the end of therapy. This underlines the effect of high tone therapy on neuropathy, as in these patients, the placebo treatment applied a few weeks before had not lead to significant changes of the symptoms.

However, the potential influence of the open-label setting on patient-related outcome results should be mentioned. Even though recent literature suggested that for example for the assessment of pain, blinded settings are favored [18], others reported no evidence of significant bias by an open-label setting [23].

In the open-label clinical observation group, significant reductions in most neuropathic symptoms in the NRS questionnaire were observed. The stronger effect may be explained by the larger group of patients. Alternatively, the potential influence of the open-label setting should be kept in mind, again. However, this setting reflects the daily clinical practice, in which all patients have expectations of their treatment. Therefore, these expectations could be counted to the treatment’s benefit.

Importantly, most patients had ongoing chemotherapy during their participation in the study. Still, a significant reduction in paresthesias was observed in the verum group. Therefore, HiToP might be a useful method to prevent patients from worsening symptoms due to ongoing treatment or even for early preventive treatment before significant symptoms even develop. However, more research is needed to verify this suggestion.

To date, the mechanisms of action of high tone therapy have not been fully clarified yet. One way of action is neuromodulation. It may be assumed that the sensory afferent input to the dorsal root ganglions modifies the hyperexcitability of the affected sensory nerve fibers. This is of special interest to our patient group, as it is known that platin causes ganglionopathy with axonal hyperexcitability [1]. Moreover, HiToP also activates motor nerve fibers and leads to muscle contraction, which modifies muscle tone and may have an additional analgetic effect.

Another way of action is the promotion of attachment and differentiation of hematopoietic stem cells were reported in diabetics [8]. In addition, neovascularization, stronger microcirculation, increase in blood flow, and improved endothelial function are discussed [5, 8, 10, 24, 35].

Some limitations of this study need to be mentioned. First, the sample size was small, as numerous patients needed to be excluded after screening. However, it was still a randomized double-blind placebo-controlled evaluation and despite the small sample, significant alleviations in neuropathy symptoms were observed. Second, the CIPN20 scales did not reflect the changes observed in the NRS questionnaire. Still, the CIPN20 has only four response options, whereas the NRS questionnaire offered more gradations. Possibly, the higher number of options in the NRS questionnaire could have made it easier to report changes in the symptoms.

Future studies should confirm the effect of high tone therapy on chemotherapy-induced peripheral neuropathy in different patient groups with larger samples. In addition, comparative analyses with other therapies (e.g., TENS) should be conducted. Moreover, early high tone therapy interventions to prevent chemotherapy-induced peripheral neuropathy should be investigated as well as different treatment regimens (e.g., frequency, duration, and electrode positions).

In summary, to the authors’ knowledge, this pilot evaluation is the first report on the effect of high tone therapy on chemotherapy-induced peripheral neuropathy. The treatment was safe and well-tolerated; no adverse events and especially no serious adverse events were reported. Significant reductions of paresthesia intensity and mental stress due to paresthesias were observed in the verum group but not in the placebo group. Based on this evaluation, high tone therapy seems to be a safe and effective option for the treatment of chemotherapy-induced peripheral neuropathy.

### Supplementary Information

Below is the link to the electronic supplementary material.

Supplementary file1 (DOCX 15 KB)

Supplementary file2 (DOCX 14 KB)

Supplementary file3 (DOCX 14 KB)

## Data Availability

The datasets generated during and/or analyzed during the current study are not publicly available but are available from the corresponding author on reasonable request.
