# Supplementary material for: Home-based high tone therapy may alleviate chemotherapy-induced neuropathic symptoms in patients with colorectal cancer: A randomized double-blind placebo-controlled pilot evaluation
Source: Support Care Cancer. 2024 Jan 27;32(2):134. doi: 10.1007/s00520-024-08331-7 (PMC10821972; doi:10.1007/s00520-024-08331-7)
Supplement: Supplementary file 1 — Supplementary file1 (DOCX 15 KB) [file 520_2024_8331_MOESM1_ESM.docx]

|  | Placebo (n=7) changes 1^st^ week of therapy | Verum (n=7) changes 1^st^ week of therapy | Placebo (n=7) changes 2w follow-up | Verum (n=7) changes 2w follow-up | F-value changes 1^st^ week of therapy (interaction with group) | p-value changes 1^st^ week of therapy (interaction with group) | F- value changes 2w follow-up (interaction with group) | p-value changes 2w follow-up (interaction with group) |
| --- | --- | --- | --- | --- | --- | --- | --- | --- |
| Intensity of paresthesias | -1.8 (-3.7-0.1) | -0.9 (-2.3-0.6) | -0.9 (-2.1-0.2) | -2.3 (-3.9--0.7) | 0,112 | n.s. | 3.272 | n.s. |
| Mental stress due to paresthesias | -1.6 (-3.9-0.6) | -1.3 (-2.4--0.2) | -0.5 (-1.9-0.9) | -2.6 (-4.6--0.6) | 0,188 | n.s. | 5.118 | 0.043 |
| Intensity of pain | 0.1 (-2.2-2.4) | -0.3 (-1.8-1.2) | 1.2 (-1.6-4.0) | -0.3 (-2.4-1.9) | 0,002 | n.s. | 1.288 | n.s. |
| Mental stress due to pain | 0.2 (-1.9-2.3) | -0.7 (-2.3-0.9) | 1.5 (-1.5-4.5) | -0.7 (-2.8-1.3) | 0,002 | n.s. | 2.576 | n.s. |
| Intensity of tightness | 0.2 (-2.5-3.0) | 0.7 (-2.7-4.1) | 0.4 (-2.5-3.2) | 0.9 (-2.8-4.5) | 0,218 | n.s. | 0.081 | n.s. |
| Mental stress due to tightness | -0.2 (-3.6-3.2) | 0.3 (-3.9-4.5) | -0.1 (-3.7-3.5) | 0.4 (-4.0-4.9) | 0,132 | n.s. | 0.053 | n.s. |
| Intensity of cramps | -1.2 (-3.0-0.6) | 0.9 (-0.6-2.3) | -0.1 (-2.5-2.4) | 0.3 (-0.5-1.0) | 0,002 | n.s. | 0.139 | n.s. |
| Mental stress due to cramps | -0.8 (-2.3-0.7) | 1.0 (-0.7-2.7) | 0.4 (-2.0-2.7) | 0.4 (-0.4-1.2) | 0,016 | n.s. | 0.006 | n.s. |

Supplementary table 3: Changes in NRS scores from baseline until the first week of therapy and from baseline until the two-week follow-up after the end of therapy, respectively. F-values under consideration of interactions with the groups were calculated.
