# Supplementary material for: Home-based high tone therapy may alleviate chemotherapy-induced neuropathic symptoms in patients with colorectal cancer: A randomized double-blind placebo-controlled pilot evaluation
Source: Support Care Cancer. 2024 Jan 27;32(2):134. doi: 10.1007/s00520-024-08331-7 (PMC10821972; doi:10.1007/s00520-024-08331-7)
Supplement: Supplementary file 2 — Supplementary file2 (DOCX 14 KB) [file 520_2024_8331_MOESM2_ESM.docx]

|  | Opt in (n=6) baseline | Opt in (n=6) change | p-value |
| --- | --- | --- | --- |
| Intensity of paresthesias | 5.66 (3.33-8.00) | -1.5 (-2.72--0.28) | 0.03 |
| Mental stress due to paresthesias | 5.50 (2.91-8.09) | -1.17 (-2.34-0.00) | 0.058 |
| Intensity of pain | 3.83 (0.71-6.96) | -0.16 (-1.50-1.16) | n.s. |
| Mental stress due to pain | 4.0 (0.65-7.35) | -0.67 (-2.73-1.40) | n.s. |
| Intensity of tightness | 2.33 (0.00-4.67) | -0.66 (-4.34-3.00) | n.s. |
| Mental stress due to tightness | 2.33 (0.00-4.67) | -0.83 (-4.49-2.82) | n.s. |
| Intensity of cramps | 2.33 (0.00-4.92) | -1.66 (-4.09-0.76) | n.s. |
| Mental stress due to cramps | 2.33 (0.00-4.92) | -1.66 (-0.09-0.76) | n.s. |

Supplementary table 2: Baseline values and changes in PNP symptoms in the numeric rating scale (NRS) in the opt in group from the start of the opt in therapy (=baseline) until the end of the opt in therapy.
