# Supplementary material for: Home-based high tone therapy may alleviate chemotherapy-induced neuropathic symptoms in patients with colorectal cancer: A randomized double-blind placebo-controlled pilot evaluation
Source: Support Care Cancer. 2024 Jan 27;32(2):134. doi: 10.1007/s00520-024-08331-7 (PMC10821972; doi:10.1007/s00520-024-08331-7)
Supplement: Supplementary file 3 — Supplementary file3 (DOCX 14 KB) [file 520_2024_8331_MOESM3_ESM.docx]

|  | Open label (n=29) baseline | Open label (n=29) change | p-value |
| --- | --- | --- | --- |
| Intensity of paresthesias | 6.83 (4.59-9.06) | -2.59 (-4.81--0.36) | <0.001 |
| Mental stress due to paresthesias | 6.76 (3.81-9.71) | -2.69 (-4.84--0.53) | <0.001 |
| Intensity of pain | 4.52 (0.78-8.26) | -2.79 (-5.88-0.29) | <0.001 |
| Mental stress due to pain | 4.55 (0.70-8.40) | -2.24 (-5.26-0.78) | <0.001 |
| Intensity of tightness | 3.62 (0.00-7.24) | -0.90 (-2.85-1.06) | 0.02 |
| Mental stress due to tightness | 3.45 (0.00-7.18) | -0.60 (-2.87-1.69) | n.s. |
| Intensity of cramps | 2.38 (0.94-5.70) | -1.41 (-4.09-1.27) | 0.008 |
| Mental stress due to cramps | 2.24 (0.00-5.51) | -1.21 (-3.91-1.50) | 0.023 |

Supplementary table 3: Baseline values and changes in PNP symptoms in the numeric rating scale (NRS) in the open label group from baseline until the end of therapy.
